# Verbal and novel multisensory associative learning in adults

**DOI:** 10.12688/f1000research.2-34.v2

**Published:** 2013-05-28

**Authors:** Joanne M Fifer, Ayla Barutchu, Mohit N Shivdasani, Sheila G Crewther

**Affiliations:** 1 School of Psychological Science, La Trobe University, Bundoora, 3086, Australia; 2 Bionics Institute, Melbourne, 3002, Australia; 3 Florey Institutes of Neuroscience and Mental Health, University of Melbourne, Melbourne, 3010, Australia

## Abstract

To date, few studies have focused on the behavioural differences between the learning of multisensory auditory-visual and intra-modal associations. More specifically, the relative benefits of novel auditory-visual and verbal-visual associations for learning have not been directly compared. In Experiment 1, 20 adult volunteers completed three paired associate learning tasks: non-verbal novel auditory-visual (novel-AV), verbal-visual (verbal-AV; using pseudowords), and visual-visual (shape-VV). Participants were directed to make a motor response to matching novel and arbitrarily related stimulus pairs. Feedback was provided to facilitate trial and error learning. The results of Signal Detection Theory analyses suggested a multisensory enhancement of learning, with significantly higher discriminability measures (d-prime) in both the novel-AV and verbal-AV tasks than the shape-VV task. Motor reaction times were also significantly faster during the verbal-AV task than during the non-verbal learning tasks.  Experiment 2 (n = 12) used a forced-choice discrimination paradigm to assess whether a difference in unisensory stimulus discriminability could account for the learning trends in Experiment 1. Participants were significantly slower at discriminating unisensory pseudowords than the novel sounds and visual shapes, which was notable given that these stimuli produced superior learning. Together the findings suggest that verbal information has an added enhancing effect on multisensory associative learning in adults

## Introduction

Effective perception of our everyday environment requires that specific associations are learnt between the multiple representations of objects and events that occur across different sensory modalities. Such multisensory associative learning, particularly between the auditory and visual modalities, plays an important role in many social and cognitive processes, including object identification as well as lexical and semantic processing ^1– 3^. Multisensory processing is known to facilitate many aspects of information processing, resulting in faster motor responses ^4^ and increased visual perceptual sensitivity ^5^. Indeed, Barutchu *et al.* ^6^ recently demonstrated that superior auditory-visual multisensory abilities were associated with above average general intellectual abilities in children. With regard to learning, stimuli that are initially encountered as concurrent and semantically congruent auditory-visual events are more reliably remembered than those encountered separately in the auditory or visual modalities ^7, 8^. Recent studies also suggest that sound modulates visual learning ^9, 10^, and that auditory-visual training compared with visual or auditory training alone can lead to marked improvements in learning ^11, 12^. Not unexpectedly, learning by experience and prior knowledge have also been shown to influence multisensory processes at both a behavioural and neural level ^1, 13– 19^.

Surprisingly few studies have investigated the differences in learning patterns within and across sensory modalities. Tanabe *et al.* ^20^ contrasted the learning of cross-modal and intra-modal associations in an fMRI investigation. They assessed feedback-based trial-by-trial learning of sequentially presented novel cross-modal and intra-modal pairs in a delayed match-to-sample task. In their study, no significant differences between auditory-visual and visual-visual learning were found. On the other hand, Seitz *et al.* ^12^ demonstrated that auditory-visual pairs were learnt to a significantly greater degree than intra-modal (auditory-auditory and visual-visual) pairs in a statistical learning task. However, the cross-modal advantage may have resulted from the method of presentation of stimuli whereby auditory-visual pairs were presented simultaneously but intra-modal pairs were presented sequentially. This systematic variation in temporal disparity of stimulus presentation may have influenced the results.

Other empirical evidence indicates that concurrently presented familiar cross-modal stimulus pairs with verbal components can influence both learning and multisensory processes ^1, 14, 15, 21^. Indeed, verbal stimuli have been shown to facilitate many cognitive processes, including object categorisation and localisation ^22, 23^. This raises the question of whether visual-verbal learning may lead to behavioural advantages when compared with non-verbal auditory-visual learning in adults. The association and transfer of visual and verbal information are of particular relevance, as such associations are known to play an important role in the acquisition of complex cognitive tasks, such as reading ^24– 26^. Furthermore, in infants, linguistic material such as nonsense words can enhance associations between stimuli and contribute to categorical learning ^27, 28^. However, to the best of our knowledge, no study has directly contrasted the learning of visual-verbal associations with non-verbal auditory-visual associations in adults.

## Experiment 1

The current study addressed two aspects of multisensory associative learning. Firstly, we aimed to evaluate performance differences on analogous intra-modal and multisensory learning tasks. Secondly, we further aimed to compare verbal and non-verbal multisensory associative learning. Three paired associate learning tasks were created: novel non-verbal auditory-visual (employing “novel sounds”; novel-AV), verbal-visual (verbal-AV), and visual-visual (shape-VV). We used a trial-and-error learning paradigm based on that of Tanabe *et al.* ^20^, but with concurrently presented stimuli, in order to examine differences in associative learning between multisensory and intra-modal stimulus pairs under simultaneous conditions. The novel-AV and shape-VV tasks utilised non-verbal stimuli, whereas the verbal-AV task included auditory pseudowords to evaluate the impact of semantically novel, yet phonetically familiar, auditory stimuli on learning. Learning performance and task differences were evaluated by analysing changes in accuracy using signal detection theory (d-prime) and motor reaction times (MRTs).

## Materials and methods

### Participants

Twenty right-handed adults (9 males), between 18 and 35 years of age (mean = 24.87 years, SD = 3.54) were recruited. All participants spoke English as a first language and were screened to ensure auditory detection thresholds and vision (distance, near and colour) were within the normal or corrected to normal ranges. Participants reported no history of neurological or psychological disorders. Estimated Full Scale IQ scores on the Wechsler Test of Adult Reading ^29^ were above the 10 ^th^ percentile for all participants except one adult, whose results were excluded from further analyses. Written informed consent was obtained from all participants. Ethics approval was obtained from the La Trobe University Human Research Ethics Committee, Bundoora, and The Royal Victorian Eye & Ear Hospital Human Research Ethics Committee, Melbourne.

### Associative learning tasks

**Figure 1.**
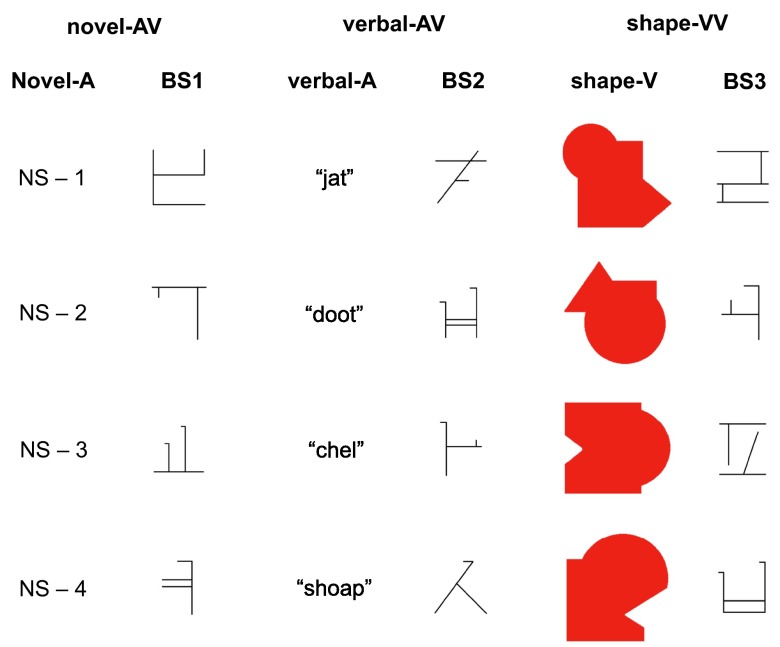
Auditory and visual stimuli of associative learning tasks. Auditory and visual stimuli for the novel sound-visual (novel-AV), the verbal-visual (verbal-AV), and the visual-visual (shape-VV) associative learning tasks.

Four novel and arbitrarily related stimulus pairs were created for each learning task (novel-AV, verbal-AV and shape-VV). All auditory and visual stimulus pairs were presented well above threshold, and were therefore easily detectable. Visual black symbols (BS) of 3 degrees of visual angle, composed using black lines against a white background (see Figure 1: BS1, BS2, BS3 for examples), were presented at participants’ central fixation point on a 22-inch cathode-ray-tube (CRT) monitor (positioned at a distance of 1 meter from participants). These visual symbols were paired either with novel sounds that were unfamiliar and could not be vocalised (novel-AV), verbal pseudowords (verbal-AV), or other visual symbols (shape-VV) (see Figure 1 for an illustration of all stimuli used).

For the novel-AV task, novel non-speech auditory sounds were digitally created (sampling rate = 48.8 kHz, duration ~620 ms, 5 ms rise-fall time), consisting of combinations of four amplitude-modulated tones using different carrier and modulation frequencies. In the novel-AV task, for each novel sound, the carrier frequencies ranged between 400–480 Hz (novel sound 1; NS-1), 1000–1350 Hz (NS-2), 3000–3200 Hz (NS-3) and 250–4500 Hz (NS-4). Modulation frequencies were either kept at 3 Hz (NS-1), 10 Hz (NS-2), 0.5 Hz (NS-3) or 6 Hz (NS-4). In addition to the amplitude modulated (AM) tones, two pure tones of 250 Hz and 450 Hz were also added to NS-4. In the verbal-AV task, four single syllable pseudowords (matched for the duration of ~620 ms) pronounced by a young female adult were used (“jat”, “doot”, “chel” and “shoap”). Each pseudoword began with a different consonant to emphasise stimulus onset. Pseudowords were pre-recorded in a sound attenuated recording booth (sampling rate = 48.8 kHz). All verbal and novel-sounds were presented at 69 dB sound pressure level (SPL) using closed headphones. The shape-VV task employed a second visual stimulus, which was a novel solid red shape constructed by overlaying rectangular, triangular and circular shapes in different combinations (see Figure 1, shape-V). In this task the black symbol was presented superimposed and contained within the red shape such that both were presented at the participant’s central fixation point. All stimulus pairs were presented concurrently for the duration of ~620 ms with simultaneous onset and offset times.

Sound files used in the experiments Sounds of pseudowords (chel, doot, jat and shoap) as well as carrier and modulation frequencies used in the experiments. Carrier frequencies: Novel sound 1 (NS-1) = 400-480 Hz. Novel sound 2 (NS-2) = 1000-1350 Hz, Novel sound 3 (NS-3) = 3000-3200 Hz. Novel sound 4 (NS-4) = 250-4500 Hz. Modulation frequencies were either kept at 3 Hz (NS-1), 10 Hz (NS-2), 0.5 Hz (NS-3) or 6 Hz (NS-4)

**Figure 2.**
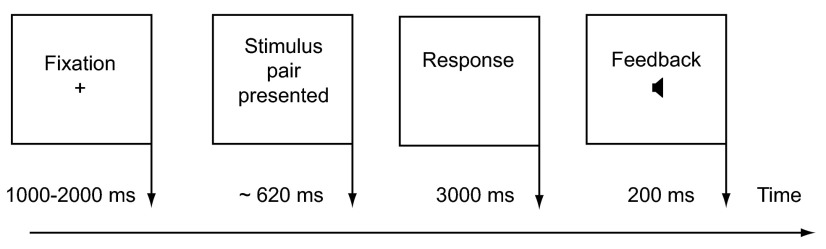
Example trial of the associative learning task. Temporal sequence of a single experimental paired associate learning trial.

Participants were required to learn the associations between the stimulus pairs via trial and error. Figure 2 presents a schematic diagram of the temporal sequence of a single trial. For each task, matching stimuli were four specific pairings of stimuli, out of a possible 16 stimulus combinations. The non-matching stimuli comprised the 12 other possible pairings of the stimuli. Each learning task was administered as four blocks of 32 trials (128 total trials). In 50% of the trials, matching pairs were presented, and the remaining trials consisted of non-matching stimuli. The presentation of stimulus pairs within each block was pseudorandom, such that each block consisted of 16 presentations of matching stimuli with each stimulus pair presented four times. Participants were instructed to make a button response with their right index finger when a matching stimulus pair was detected. A ‘no response’ was deemed to be an indication that the participant did not consider the stimuli to be a matched pair. Participants were allowed 3000 ms to respond, after which feedback was provided after every trial using an ascending tone burst (for a correct response) or descending tone burst (for an incorrect response), presented for a duration of 200 ms. The feedback was followed by an inter-stimulus interval, which randomly varied between 1000 to 2000 ms.

### Procedure

Participants completed the three tasks in a quiet room. Testing took place over one or two sessions, and the total test time per participant was approximately 2 hours. When two or more learning tasks were completed in a single session, a period of at least 45 minutes was implemented in-between tasks to maximise concentration for the subsequent learning task. During this time, the auditory and visual screening tasks were completed. The order in which the three learning tasks (novel-AV, verbal-AV, and shape-VV) were administered was counterbalanced across participants.

A practice task was employed prior to each experimental learning task. The practice task was analogous to the proceeding experimental task; however, there were only three stimulus pairs (as opposed to four), and the stimuli were not abstract (e.g., visual stimuli were images of a square, circle and triangle rather than unfamiliar shapes). Participants were encouraged to ask questions about the practice task and up to 60 trials were administered until such time as the participant demonstrated understanding of the task requirements. In addition, immediately before the experimental tasks began, participants were presented once with each of the eight individual stimuli that made up the four stimulus pairs. This familiarisation process ensured that participants were aware of the characteristics of each stimulus, but did not receive any information regarding whether they were matching or not. Participants were instructed that during the experimental task they would be shown novel symbols, for which they would need to learn the associated pseudowords (verbal-AV), sounds (novel-AV), or shapes (shape-VV). Each of the four blocks within each task was of approximately 4 minutes duration, and participants were offered a short break of up to 1 minute between blocks.

### Data analysis

Accuracy data and motor reaction times (MRTs) were recorded. An error on a matching trial was a failure to make a motor response (i.e., miss). A motor response to a non-matching trial reflected a false alarm. Only the first 120 trials were analysed, as some participants failed to complete the entire set of 128 trials due to computer failure (only one participant on the verbal-AV condition was affected). All participants completed the first 120 trials that were analysed.

To visualise the different patterns of learning across trials for each task and stimulus pair type, “five-point moving average” analyses were employed to construct learning curves consisting of 116 overlapping windows moving in one trial steps, which were averaged across participants. Sets of five trials were used because preliminary analysis revealed that considerable learning occurred early in the experiment, and small window lengths allowed for optimal visualisation of learning effects.

Signal Detection Theory (STD) and measures of discriminability (d-prime) were used to assess changes in learning trends for the three stimulus pair types. The discrimination statistic, usually known as d-prime, is a measure of an individual’s ability to distinguish true signals from noise ^30, 31^. It incorporates true responses and false alarms to minimize the effects of response bias to target and non-target stimuli. The d-prime statistic was calculated as the difference between Z scorers for hit rates and false alarms [i.e., d-prime = Z(hit rate)-Z(false alarm rate)] for the total 120 trials and for the three stimulus pair types. The differences between each pair type were analyzed using a one-way repeated measures ANOVA. We were also interested in the ability to discriminate pairs as learning occurred across time, therefore data was subdivided into blocks of five trials each; there were a total of 24 successive blocks. Preliminary analyses revealed that d-prime values for block 8 and all subsequent blocks violated normality, thus analysis of the effects of task and stimulus pair type across the first 7 blocks were assessed using a 3 (task: novel-AV, verbal-AV, shape-AV) × 7 (block: 1, 2, 3, 4, 5, 6, 7) repeated measures ANOVA.

**Figure 3.**
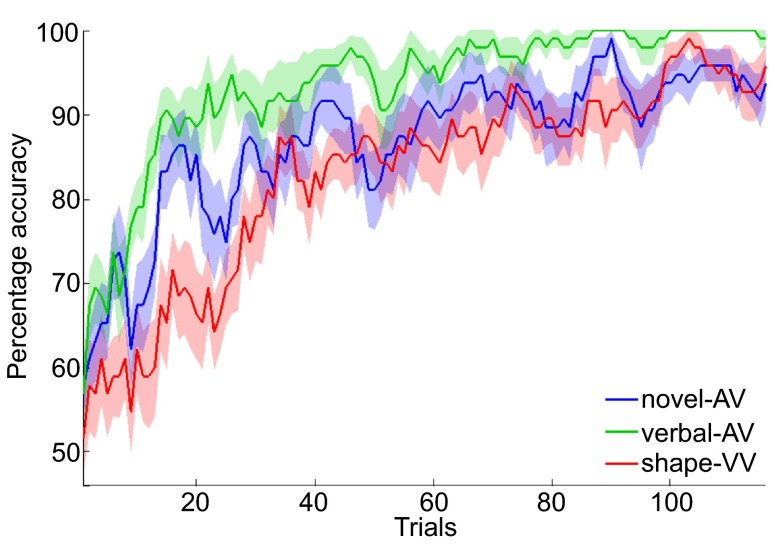
Percentage accuracy for the associative learning tasks. Moving average of mean percentage accuracy for trials on the novel-sound auditory-visual (novel-AV), the verbal-visual (verbal-AV), and the visual-visual with red shapes (shape-VV) learning tasks. Shaded areas depict SEM along the moving average.

Changes in MRTs with learning were also investigated. Based on learning trends observed in the moving average learning curves (see Figure 3), the first 20 matching trials were defined as the “learning” phase and final 20 target trials were defined as the “learnt” phase (please note that non-target stimuli did not require a response, hence could not be included in this analysis). Differences in learning between tasks were assessed using a 3 (task: novel-AV, verbal-AV, shape-VV) × 2 (phase of learning: learning, learnt) repeated measures ANOVA.

All significant main effects obtained in ANOVA analyses were followed up with post-hoc pairwise comparisons using Tukey corrections. For all statistical tests, alpha level was set at 0.05.

## Results

### Analysis of learning across time

Participants demonstrated learning in all three experimental tasks (see Figure 3). It can be seen that under all conditions, initial performance was close to chance levels, and accuracy rates above 90 percent were attained on each task. The verbal-AV task was consistently performed with greater accuracy than both non-verbal tasks, with a steeper trajectory of learning and with accuracy rates reaching ceiling performance during the second half of the experiment. Of the non-verbal tasks, the novel-AV task tended to be performed with slightly greater accuracy than the shape-VV task. This novel-AV superiority was primarily evident at early learning stages; novel-AV and shape-VV accuracy rates were more similar from trial 50 onwards.

**Figure 4.**
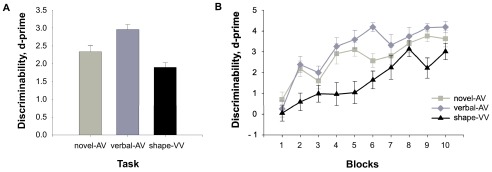
Discriminability measures for associative learning tasks. **A**. Overall mean discriminability (d-prime) (± SEM) for the novel-sound auditory-visual (novel-AV), the verbal-visual (verbal-AV), and the visual-visual with red shapes (shape-VV) learning tasks. **B**. Discriminability (d-prime) first 10 blocks of trials on the novel-AV, the verbal-AV, and the shape-VV learning tasks. Each block comprised 5 consecutive trials.

Similar effects of task were evident when d-prime measures were considered. Overall mean discriminability (d-prime) significantly differed across all stimulus conditions, (see Figure 4A), *F*(2, 38) = 16.77, *p* < 0.001. The mean d-prime value for the verbal-AV task was significantly higher than that for the novel-AV, both of which were significantly higher than the shape-VV task.

Analyses of discriminability across early blocks ( Figure 4B) revealed that there were no discernable differences in the patterns of learning for each task, as the interaction between task and block was not significant, *F*(12, 216) = 1.54, *p* > 0.01. However, it was found that overall discriminability measures for the three task types significantly differed across these first 7 blocks, *F*(2, 216) = 22.40, *p* < 0.001. Performance rates were significantly different between all tasks, with verbal-AV producing the greatest discriminabilty and shape-VV the poorest. In addition, with all tasks considered, mean d-prime measures significantly increased between the first two blocks, and then significant increases in discriminability occurred at block 5 before plateauing thereafter *F*(6, 216) = 13.45, *p* < 0.001.

Experiment 1 raw data Accuracy and motor reaction times (MRT) data for all participants on novel-AV (“AV”), shape-VV (“VV”), and verbal-AV (“Vver”) learning tasks. The values in each column denote:
Stimulus type: 1-4 = target; 5-16 = non-target
Response/Expected response: 1 = press; 0 = no press
Accuracy: 0 = correct; 1 = incorrect

### Motor reaction time analyses

**Figure 5.**
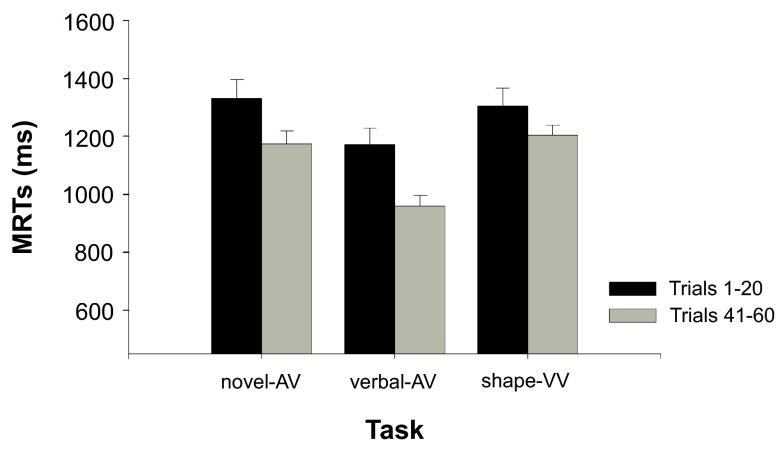
Reaction times for associative learning tasks. Average motor reaction times (MRTs) (± SEM) for the learning phase (Trials 1–20) and the learnt phase (Trials 41–60) in the novel-sound auditory visual (novel-AV), verbal-visual (verbal-AV), and visual-visual (shape-AV) learning tasks.

As can be observed in Figure 3, moving averages of percent accuracy show steep learning trends within the first 20 trials with performance almost reaching ceiling levels by the end of the first 60 trials. We therefore further investigated differences in motor responses during the learning stage (first 20 trials) and the late learning stage (41–60 trials) for matching stimuli (note that non-matching stimuli did not require a response, hence, non-matching trials could not be included in the MRT analyses). A significant interaction effect between learning phase and task type was found, *F*(2, 36) = 3.56, *p* < 0.05, partial *η ^2^* = 0.17. At both learning stages, the MRTs for the verbal-AV condition were significantly faster than for the novel-AV and shape-VV tasks (see Figure 5); the MRTs for novel-AV and shape-VV tasks did not differ significantly from each other in the first 20 target trials. Furthermore, the rate of change in MRTs was significantly greater for verbal-AV trials compared with shape-VV trials. The change in MRTs for novel-AV trials did not significantly differ from either verbal-AV or shape-VV trials.

## Discussion

The results of Experiment 1 demonstrated a significant advantage for the learning of both verbal and non-verbal auditory-visual stimulus associations over intra-modal visual-visual associations. In the verbal-visual task, associated stimuli were also identified faster. Whilst these results represent potentially important and compelling findings, it remained difficult to delineate the underlying factors causing the learning differences. The most pressing factor was the possible confounding influence of differences in stimulus discriminability at a unisensory level. A follow-up experiment evaluated whether unequal unisensory stimulus discriminability could explain differences in learning trends between the verbal-AV, novel-AV and shape-VV tasks.

## Experiment 2

The aim of Experiment 2 was to investigate whether the unisensory auditory and visual stimuli employed in the associative learning paradigms differ in discrimination difficulty. The auditory and visual stimulus sets from the learning tasks were tested in six separate forced-choice discrimination tasks (see Figure 1 for an illustration of all 6 stimulus sets). Here we investigated discrimination difficulty across the three differing task-specific stimuli: novel auditory stimuli (novel-A), verbal auditory stimuli (verbal-A), and red-shape visual stimuli (shape-V), in addition to the three sets of visual black symbols that were paired with: BS1, BS2 and BS3, respectively.

## Method

### Participants

Participants were 12 adult volunteers (6 males), aged between 18 and 35 years ( *M* = 29.55, *SD* = 3.43). Participants who took part in the current control study did not participate in the learning task. All participants were right handed, had normal or corrected to normal vision, normal hearing and no prior history of neurological or psychiatric disorders.

### Stimuli and discrimination tasks

The forced-choice discrimination tasks utilised the six stimulus sets that were employed in the three learning tasks in Experiment 1: novel sounds (novel-A), verbal pseudowords (verbal-A), red shapes (shape-V), BS1, BS2 and BS3 (see Figure 1 for an illustration of stimuli). There were four stimuli in each discrimination task, and for each task, one of the four stimuli was randomly assigned as the “target” stimulus, and the remaining three stimuli as “non-target” stimuli. The target stimuli were alternated via counterbalancing across participants such that each stimulus acted as the target stimulus on an equal number of occasions.

Stimuli were presented individually in the discrimination tasks. Visual stimuli were presented at central fixation on a 17-inch laptop screen at three degrees of visual angle. Auditory stimuli were presented via closed headphones. Both the auditory and visual stimuli were presented for ~620 ms duration. All other stimulus parameters for the novel sounds, verbal psuedowords, red shapes and visual black symbols (BS1, BS2 and BS3) were same as in Experiment 1.

For each task, participants were presented with 80 stimuli in two blocks of 40 trials each. The inter-stimulus interval (ISI) randomly varied between 1000–1500 ms during which a central fixation point was presented. Consistent with the learning tasks in Experiment 1, stimuli were presented in a pseudorandom order with a 0.5 target probability. The remaining stimuli consisted of non-targets with an equal probability of each type. The same stimulus was not presented for more than two consecutive trials. Participants were asked to indicate whether the stimuli were targets or non-targets by pressing buttons on a keyboard. A feedback tone pip (700 Hz, 122 ms in duration) was provided at the end of a trial if the response was incorrect.

### Procedure

All participants completed the six discrimination tasks. The order of task administration was counterbalanced across participants to negate any order or practice effects. Participants were instructed to press the left arrow key on a standard keyboard with their index finger in response to a target, and the right arrow key with their middle finger in response to any non-target stimuli. Participants were instructed to respond as quickly and accurately as possible. A feedback tone pip was provided when the participant’s response was incorrect; no feedback was provided for a correct response. Prior to testing, participants were given up to 20 trials as practice during which they were familiarised with the target stimulus and were able to practice performing the task.

### Data analysis

Percentage error and mean MRTs for target and non-target stimuli were calculated. All participants completed 80 trials; whilst a lower-bound response inclusion criterion of 150 ms was set to control for response anticipation, there were no responses of this nature, so no data was excluded on this basis.

For MRTs, two repeated measures ANOVAs were used to analyse differences between novel sounds, verbal pseudowords and visual shapes, and the three black symbol sets separately. Any significant effects were followed up with Tukey post-hoc tests.

## Results

The percentage error rates for all discrimination paradigms were very low, averaging below 6% error rates (see Table 1). Many participants did not make discrimination errors, leading to violations of normality. Therefore, accuracy measures were not subjected to further data analyses.

**Table 1.** Mean and median (+ SD) percent error rate for target and non-target (other) trials on the six discrimination tasks: novel auditory sounds (novel-A), verbal auditory sounds (verbal-A), visual red shapes (shape-V), and the three sets of black symbol sets (BS1, BS2 and BS3).

Task Specific Stimuli						Black Symbol Sets			
		*Mean*	*Med*	*SD*			*Mean*	*Med*	*SD*
Novel-A	Target	4.48	2.50	4.38		BS1	3.54	2.5	4.32
Verbal-A	Target	2.92	2.50	2.57		BS2	4.27	3.75	3.59
Shape-V	Target	5.10	4.38	3.82		BS3	2.50	1.25	3.59

**Figure 6.**
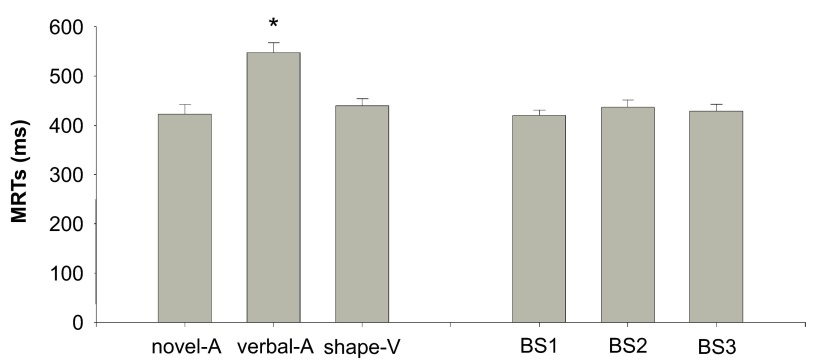
Reaction times for unisensory stimulus discrimination. Mean MRTs (+SEM) for each of the discrimination tasks: novel auditory sounds (novel-A), verbal auditory sounds (verbal-A), visual red shapes (shape-V), and the black visual symbol sets (BS1, BS2 and BS3). * *p* < 0.05.

The MRTs for the novel-A, verbal-A and shape-V discrimination stimuli significantly differed from each other (see Figure 6), *F*(2, 22) = 26.58, *p* < 0.001, partial *η ^2^* = 0.71. MRTs were significantly slower for verbal auditory stimuli (verbal-A) than both novel auditory (novel-A) and visual red shape (shape-V) stimuli. However, the MRTs for novel-A and shape-V stimuli did not significantly differ from each other. No significant differences were found between MRTs for the three black symbol sets, *F*(2, 22) = 1.36, *p* > 0.05, partial *η ^2^* = 0.11.

Experiment 2 raw data Accuracy and motor reaction times (MRT) data for all participants on Novel-A (“NovelSounds”), Verbal-A (“VerbalSounds”), Shape-V (“RedShapes”), and three black shapes (BS) (LineSet1, 2, and 3) discrimination tasks. The values in each column denote:
Stimulus type: 1 = target; 2, 3, 4 = non-target
User Response: 1 = respond target; 2 = respond non-target; 999 = invalid response (both buttons pressed in response to the stimuli)
Accuracy: 1 = correct; 0 = incorrect

## Discussion

All auditory and visual stimuli were relatively easy to discriminate. Differences in discrimination difficulty were minimal with accuracy measures being at ceiling for all stimulus types. Although motor reaction times did not significantly differ between the black symbol sets, motor responses were significantly slower for verbal stimuli compared with the novel auditory stimuli and the red shape stimuli. This finding is consistent with prior studies suggesting that verbal stimuli require longer processing times than visual pictures ^19^. Nevertheless, the high accuracy rates for all stimulus sets suggest that any differences in discrimination difficulty between stimulus sets are relatively small, with verbal stimuli being slower to process.

It may be argued that relative differences in the memorability of the unisensory stimuli, in addition to their discriminability, may have influenced performance in the three learning tasks in Experiment 1. However, successful performance of the current discrimination task in this experiment necessitated memory processing. An accurate and speedy response to the presented stimulus required the comparison of that stimulus to a memory trace of the “target” stimulus. Therefore, the lack of significant differences between discrimination accuracy and MRTs to the auditory and visual stimuli suggests that the stimuli did not differ in their discriminability or memoriability.

## General discussion

The current results provide new evidence regarding the differences between multisensory and intra-modal learning, with more accurate performances and faster learning on the verbal-visual and novel auditory-visual tasks compared with the visual-visual task, suggesting that multisensory processing facilitates associative learning. The enhanced speed of response during the learning of verbal-visual associations compared with non-verbal stimulus associations suggest that verbal information further facilitates the associative learning process. The results of Experiment 2 suggested that the obtained task-related learning effects represented differences in associative learning rather than relative differences in stimulus discriminability between auditory and visual stimuli.

An enhancement in the learning of multisensory stimuli relative to intra-modal stimuli is not unexpected given that multisensory processing has long been known to facilitate information processing ^32^. This facilitative effect has been shown to be substantially greater for multisensory stimulus combinations than for multiple stimuli within a single modality ^33^. Not only do multisensory processes facilitate memory ^7^, but they have also been shown to enhance perceptual and implicit learning, and training outcomes ^11, 12, 34– 36^. Consistent with these prior findings, the results of the present study indicate that this multisensory advantage extends to paired associative learning. Novel auditory-visual and verbal-visual tasks were performed with greater levels of discrimination during learning compared with the visual-visual task. Motor responses were also faster with the learning of multisensory rather than unisensory stimuli, even though the motor reaction times did not significantly differ during early stages of learning of the novel auditory-visual and unisensory visual tasks. These significant learning results were in the context of non-significant differences in motor responses between stimuli employed in the novel-AV and shape-VV tasks.

The afore-mentioned multisensory enhancement of learning contrasted with the behavioural findings of Tanabe *et al.* ^20^. The differences between the results of our study and those of the fMRI study of Tanabe *et al.* ^20^ may be explained by the fact that they presented stimulus pairs sequentially, 15 seconds apart, whilst we presented stimulus pairs simultaneously. The temporally concurrent presentation of multisensory stimuli would maximise the likelihood that multisensory ‘integrative’ processes are engaged. Animal studies using single-cell recordings have demonstrated that up-regulation of neural activation occurs only when stimuli are presented within a temporal window of 500 ms ^37^. Similarly, behavioural facilitation in humans is observed only when auditory and visual stimuli are presented in close spatial and temporal proximity (reviewed in Spence and Squire ^38^ and Wallance *et al.* ^39^). An alternative explanation is that our concurrent presentation may have emphasised differences in salience and attentional resources within and across sensory modalities during dual stimulus presentations. Whilst concurrent stimulus presentations within the one modality (e.g., auditory-auditory or visual-visual) can have an interfering effect on stimulus perception, there is no such performance decrement when two concurrent stimuli are presented across different modalities (i.e., auditory-visual), even when stimuli are semantically incongruent ^13, 40^. Thus, the concurrent presentation of stimuli in the present study is likely to have facilitated information processing via modulation of either integrative or attentional processes, resulting in enhanced learning of novel multisensory stimulus pairs.

Whilst both novel auditory-visual and verbal-visual tasks were associated with superior learning performances compared to the visual-visual task, additional performance gains were observed in the verbal-visual task. Both d-prime analyses indicated that greater discrimination of learned pairs was achieved in the verbal-visual task relative to the novel auditory-visual task, and the MRTs were significantly faster in the verbal-visual task in Experiment 1, yet participants were significantly slower at discriminating unisensory verbal stimuli in Experiment 2. These verbal-visual findings are noteworthy, given that the verbal stimuli used in all learning tasks in the present study were novel and therefore lacked semantic content. The only familiar or categorisable aspect of the verbal stimuli was the phonetic and verbalisable nature of the pseudowords, which allow for quicker encoding and rehearsal of the auditory stimulus component ^41, 42^. The visual-verbal learning advantage was evident despite the fact that participants were significantly slower at discriminating the individual verbal stimuli than the novel sounds and visual shapes. This finding is consistent with prior studies showing that verbal information requires longer time to processes than visual pictures ^19^. Thus, the observed enhancements for verbal-visual associative learning cannot be attributed to a possible imbalance in discrimination difficulty between tasks; rather it appears that the processing speed advantage in the verbal-AV task is most likely related to a verbal-visual learning enhancement. This result suggests that verbal information is likely to play an important role in facilitating the accuracy and speed of human learning.

From an early age (and throughout life) we assign arbitrary, speech-based labels to visual stimuli encountered in the environment. Even though the verbal stimuli in this study were not known words, the task reflects a common requirement of daily life, which often involves previously non-encountered words to be learnt and associated with an object or person (e.g., surnames, technical or scientific terms). Similarly, in infants, nonsense words have been shown to enhance associations between stimuli and contribute to categorical learning ^27, 28^. Verbal stimuli have also been shown to enhance other cognitive processes in adults, such as object categorization ^22, 23^. The known role of the superior temporal sulcus (STS) in performing speech and language-based functions in addition to multisensory auditory-visual integration may suggest that it plays a role in subserving this necessary skill ^20, 43– 46^. Other studies have also shown greater levels of activation to multisensory stimuli involving phonetic elements in STS compared with novel auditory-visual stimuli ^1, 47^. Thus, it is likely that different neural networks underlie the processing of verbal-visual stimuli and non-verbal novel auditory-visual stimuli.

In conclusion, the present study revealed two major influences on paired associative learning. Firstly, improvements in accuracy and MRTs were demonstrated in multisensory learning tasks compared with the visual-visual learning task, and secondly, further gains in performance were obtained when auditory stimuli contained a verbal component. These results indicate that multisensory integration can facilitate associative learning, and provides new evidence that this facilitation may be further enhanced by verbal content.
