# Supplementary material for: Verbal and novel multisensory associative learning in adults
Source: F1000Res. 2013 May 28;2:34. Originally published 2013 Feb 7. [Version 2] doi: 10.12688/f1000research.2-34.v2 (PMC3907154; doi:10.12688/f1000research.2-34.v2)
Supplement: Peer review history

# Peer review: Referee response for version 2

**Published:** 2014-01-23

**Authors:** Trevor Hine

**Affiliations:** 1 Griffith University, Queensland, Australia

I have read this submission. I believe that I have an appropriate level of expertise to confirm that it is of an acceptable scientific standard.

# Peer review: Referee response for version 1

**Published:** 2013-03-26

**Authors:** Trevor Hine

**Affiliations:** 1 Griffith University, Queensland, Australia

Two well-run experiments: the first of which shows important differences not just in the rate of cross-modal vs uni-modal learning, but also in the processing speed involved in recognising these learned associations. The second experiment is an important control for the discriminability and processing speed for stimuli used in the first experiment. Together, this shows that the effects in the first experiment of learning simultaneously paired associations are due to genuine cross-modal  facilitation.

Other minor points:

Page 4. Calculation of d-prime does not involve just the ‘hit’ and ‘false alarm’ frequencies, but also ‘misses’ and ‘correct rejections’ frequencies.  Given this, it would be helpful if the authors made it more explicit how they calculated d-prime from your raw data.

Page 7.In the sentence  ‘No significant differences were found between MRTs for the three black symbol  sets, F(2, 22) = 1.36, p < .05, partial η2 = . 11.’ There is a typo ‘p > .05’.

I have read this submission. I believe that I have an appropriate level of expertise to confirm that it is of an acceptable scientific standard.

# Response

**Published:** 2013-04-29

**Authors:** Sheila Crewther

**Affiliations:** Psychological Science, La Trobe Univeristy, Australia

Dear Dr Hine, 

 

Thank you for picking up the typo and highlighting the ambiguity regarding d-prime. Indeed d-prime involves misses and correct rejection frequencies. In our case we calculated d-prime as the difference between 'hit' and 'false alarm' z scores [i.e, d-prime = Z(hit rate) - Z(false alarm rate)]. 

 

Regards Sheila Crewther.  PhD

# Peer review: Referee response for version 1

**Published:** 2013-02-14

**Authors:** Chris Baker

**Affiliations:** 1 Laboratory of Brain and Cognition, National Institute of Mental Health, National Institutes of Health, Bethesda, MD, USA

I have read this submission. I believe that I have an appropriate level of expertise to confirm that it is of an acceptable scientific standard.
